# Aptamer Conformation Switching-Induced Two-Stage Amplification for Fluorescent Detection of Proteins

**DOI:** 10.3390/s19010077

**Published:** 2018-12-26

**Authors:** Qiao Yu, Fenfen Zhai, Hong Zhou, Zonghua Wang

**Affiliations:** 1 Shandong Sino-Japanese Center for Collaborative Research of Carbon Nanomaterials, College of Chemistry and Chemical Engineering, Qingdao University, Qingdao 266071, China; 15071436638@163.com; 2 Shandong Provincial Key Laboratory of Detection Technology for Tumor Markers, College of Chemistry and Chemical Engineering, Linyi University, Linyi 276005, China; 15753928362@163.com; 3 Shandong Provincial Key Laboratory of Life-Organic Analysis, College of Chemistry and Chemical Engineering, Qufu Normal University, Qufu 273165, China

**Keywords:** platelet-derived growth factor, fluorescence detection, aptamer sensing, molecular beacon, isothermal amplification

## Abstract

Basing on the conformation change of aptamer caused by proteins, a simple and sensitive protein fluorescent assay strategy is proposed, which is assisted by the isothermal amplification reaction of polymerase and nicking endonuclease. In the presence of platelet-derived growth factor (PDGF-BB), the natural conformation of a DNA aptamer would change into a Y-shaped complex, which could hybridize with a molecular beacon (MB) and form a DNA duplex, leading to the open state of the MB and generating a fluorescence signal. Subsequently, with further assistance of isothermal recycling amplification strategies, the designed aptamer sensing platform showed an increment of fluorescence. As a benefit of this amplified strategy, the limit of detection (LOD) was lowered to 0.74 ng/mL, which is much lower than previous reports. This strategy not only offers a new simple, specific, and efficient platform to quantify the target protein in low concentrations, but also shows a powerful approach without multiple washing steps, as well as a precious implementation that has the potential to be integrated into portable, low-cost, and simplified devices for diagnostic applications.

## 1. Introduction

Proteins are ubiquitous in life and play critical roles in living organisms. The recognition, detection, and quantification of cancer-related protein biomarkers are of particular significance to the process of practical applications, including medical diagnosis, prevention, and treatment [1,2]. In general, the versatile method for the detection of proteins is based on the relevant antibody, with the alliance of different technologies, such as Raman scattering [3], fluorescence [4,5], electrochemical sensors [6,7], colorimetric assays [8,9], and surface plasmon resonance (SPR) [10,11], etc. Nevertheless, these methods are usually subject to limited dynamic range, multiple assay processes, enzyme labeling, and sophisticated equipment. Therefore, reliable homogeneous methods that could quantify protein biomarkers simply and rapidly, with high sensitivity, selectivity, and less time, is desirable, especially in resource-limited regions. Over the last decade, as potential next-generation biorecognition molecules, aptamers have aroused researchers’ wide interest and have been employed in various analytical applications [12,13]. Aptamers, as a single-stranded DNA or RNA molecules, can specifically bind to target small molecules, proteins, or cells with high affinity, and form distinct secondary and tertiary structures. Compared with traditional antibodies, aptamers have some advantages, such as easier artificial synthesis with very low cost, reversible thermal denaturation, high stability and high reproducibility in target recognition, ease of labeling, chemical modification and storage, chemical stability under a wide range of buffer conditions, and strong resistance to severe treatments without loss of bioactivity. Due to the rapid development of aptamer Systematic Evolution of Ligands by Exponential Enrichment (SELEX) technology and many efforts from researchers, the number of small or larger molecules with their own specific aptamer has greatly increased. These characteristics have promoted aptamers as significant and multifunctional components of signal transduction for developing sensing systems of various analytes with high selectivity.

In order to realize sensitive detection of low-abundance analytes, which play an important role in biological processes, much attention has been put on how to provide enhanced performance with ultrahigh sensitivity, coupling these aptamer-based biosensing technologies with signal amplification strategies. A series of amplification strategies or principles have been developed, such as bio-functional nanomaterials or the isothermal amplification of nucleic acids [14,15,16,17]. Among these amplification strategies, the amplification strategy assisted by isothermal amplification of oligonucleotides has already become one of the more powerful tools for efficient and smart signal amplified detection under gentle and constant temperature. Additionally, molecular beacon (MB) technology, an analytical strategy building on the principle of base pairing and Fӧrster resonance energy transfer (FRET) is widespread in preclinical medicine and biology for its wonderful specificity and sensitivity [18,19,20]. In the absence of a target, the MB has a hairpin structure with close distance between the fluorophore and the quencher, which is enough to generate FRET. Then, the fluorophore as a donor chromophore, initially in its electronic excited state, may transfer energy to the quencher, which acts as an acceptor through nonradiative dipole–dipole coupling with an extremely low background. However, with the addition of target molecules, the quencher tends to keep away from the fluorophore, arising from the formation of a rigid and stable DNA duplex; therefore, the process of FRET is hindered, and the fluorescence signal recovers eventually. 

In this manuscript, we proposed a smart aptamer sensing method for a highly sensitive and selective PDGF-BB assay through target protein-induced fluorescence changes via DNA molecules’ conformation changes and isothermal recycling amplification strategies. More importantly, the designed aptamer sensors show a simple but powerful approach without multiple washing steps, as well as a precious implement; thus, this method has the potential to be integrated into portable, low-cost, and simplified devices for diagnostic applications.

## 2. Experimental Section

### 2.1. Reagents 

Prostate-specific antigen (PSA), carcino-embryonic antigen (CEA), and bovine serum albumin (BSA) were obtained from Sigma-Aldrich (St. Louis, MO, USA). The deoxynucleotide solution mixture (dNTPs), polymerase Klenow Fragment exo- (10 U/μL) accompanied by 10× Klenow Fragment exo- buffer, and the nicking endonuclease Nt.BbvCI accompanied by 10× New England Biolabs (NEB) buffer were purchased from New England Biolabs Ltd (Beijing, China). The platelet-derived growth factor (PDGF)-BB was purchased from Peprotech (Rocky Hill, NJ, USA) and pre-constituted in 4 mM HCl (Nanjing Chemical Reagent Co., Ltd. Nanjing, China) with 0.1% bovine serum albumin (BSA). Ultrapure water (18.25 MΩ·cm, 25 °C) was used for all of the experiments. 

All other reagents of analytical reagent grade were used as received without further purification. The oligonucleotides designed in this study were synthesized by TaKaRa Biotechnology Co. Ltd. (Dalian, China), and their sequences are as follows: 

Signaling Probe: 5’-CY5-CGA CTC GTT CCT GCT CGG ATC TGA GGT GCA GTG AAA ACG AGT CG-BHQ3-3’;

DNA aptamer: 5’-TGC ACC TCA GCA GCA GGC TAC GGC ACG TAG AGC ATC ACC ATG ATC CTG CAT CCG AGC AGG AAC G-3’;

Control DNA aptamer: 5’- TGC ACC TCA GCA TTT CCG TGG TAG GGC AGG TTG GGG TGA TTT CTA TTC AAT CCG AGC AGG AAC G -3’;

Primer: 5’-CGACTCGT-3’

### 2.2. Equipment

Fluorescence spectra was obtained by a Hitachi F-7000 fluorescence spectrometer (Hitachi Ltd., Japan). Excitation and emission slits were all set for a 5.0 nm band-pass, respectively. The mixtures in square quartz cuvettes were excited at 640 nm, and the emission spectra was collected from 650 to 750 nm. The fluorescence intensity at 670 nm was used to evaluate the performances of the proposed assay strategy.

### 2.3. Procedure for Fluorescence Detection of the Platelet-Derived Growth Factor

The DNA aptamer (5 μL, 1 μM) reacted with the mixture of the PDGF-BB sample (5 μL) at 37 °C for 15 min. Then, the signaling probe (7 μL, 1 μM), primer (3 μL, 2.5 μM), 3.0× Klenow Fragment exo- buffer (3.0 μL) (KF buffer), and dNTPs (2 μL, 2 mM) were injected into the resulting solution successively. In the meantime, 1 μL of the Klenow Fragment exo- (1 U/μL) was added. Next, 3.0× Nt.BbvCI buffer (3.0 μL) (NEB buffer) and 1 μL of Nt.BbvCI (1 U/μL) was added. After being incubated at 37 °C for 2 h, the polymerization reaction was stopped by the addition of 0.5 M ethylenediaminetetraacetic acid (EDTA, 1 μL). In order to obtain information on the amount of target in the sample, 170 μL of ultrapure water was added to the resulting solution. The obtained solutions were detected by a fluorometer promptly with the λex = 640 nm (slit 5 nm) and λem= 670 nm (slit 5 nm).

## 3. Results and Discussion

### 3.1. The Design of an Amplified Aptasensing Platform for Fluorescence Detection of the Platelet-Derived Growth Factor

**Scheme 1:**
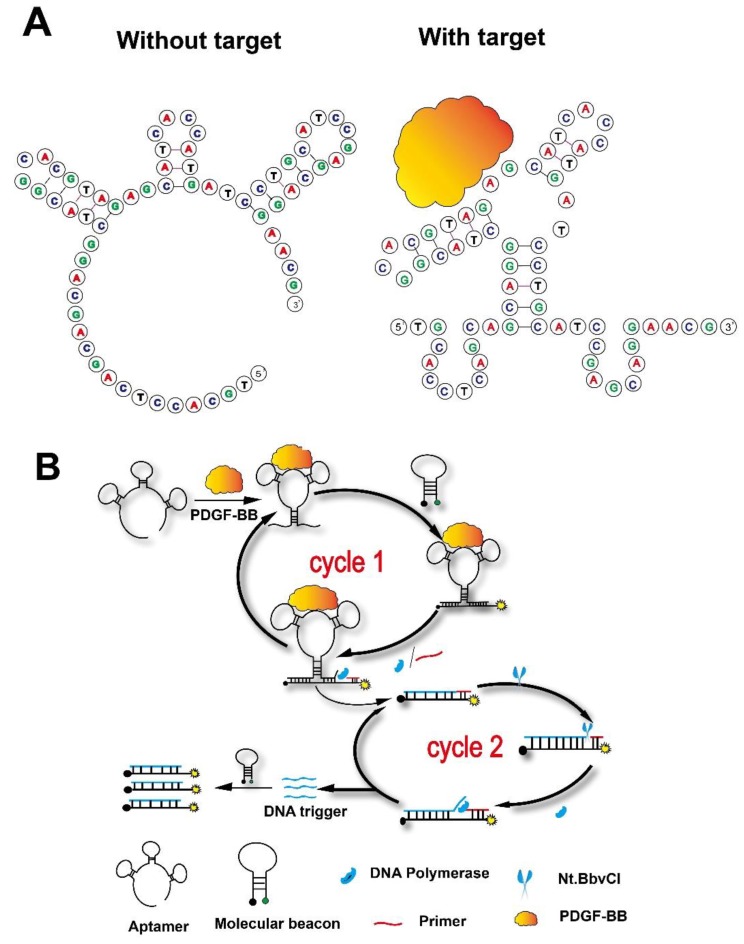
Schematics of (**A**) predicted secondary structure of the aptamer probe without (left panel) and with (right panel) target molecules. (**B**) The mechanism of an aptasensing platform combining an aptamer recognition unit and oligonucleotide-based isothermal strand displacement amplification (SDA) for fluorescence detection of the platelet-derived growth factor (PDGF-BB).

In this work, with the assistance of a molecular beacon, we designed a smart aptasensing platform, combining an aptamer recognition unit and oligonucleotide-based isothermal strand displacement amplification (SDA). The aptasensor showed excellent performance for the detection of the target protein via a two-stage amplification reaction assisted by polymerase and nicking endonuclease. Platelet derived growth factor (PDGF-BB), a cell growth and division-related protein, was chosen as a model protein to validate the concept of our engineering, owing to its association with several diseases—for instance, atherosclerosis and malignant tumors. As shown in Scheme 1, the sensing system is comprised of a DNA aptamer, assistant primer, and molecular beacon (MB), modified with fluorophore Cy5 and quencher BHQ3 at the 5′ and 3′ terminus, respectively. Herein, the sequences of the aptamer for PDGF-BB is specific, and designed according to Yu’s and Yang’s previous work [4,21]. The molecular beacon probe was designed considering part of the aptamer’s sequences as well as part of recognition sequence of nicking endonuclease Nt.BbvCI, with the help of Integrated DNA Technologies [22]. Without the existence of target PDGF-BB, the DNA aptamer is in the state of its natural secondary structure, which cannot hybridize with the MB and open it. However, the natural conformation of DNA aptamer would change into a Y-shaped complex in the solution with the presence of the target protein for the special affinity binding between the DNA aptamer and PDGF-BB. Then the Y-shaped DNA aptamer could further hybridize with the MB and form a DNA duplex, leading to the open state of the MB. Subsequently, the fluorescence originating from the MB is released, because the quencher moves far away from fluorophore Cy5. Upon the formed duplex, a primer is introduced and hybridizes with the available single-stranded domain at the 3’ end of the MB, and thus DNA polymerization reaction initiates under the condition of DNA polymerase. During this DNA polymerization reaction, MB served as a template, and the Y-shaped complex would be replaced gradually. Then this Y-shaped complex composing of the target and DNA aptamer is available again for another MB in solution and to generate further DNA polymerization reaction, and following the released Y-shaped complex, triggering the first circular amplification reaction (cycle 1). Moreover, in the presence of nicking endonuclease, the generated double-stranded DNA in cycle 1 could further increase another nucleic acid recycling amplification reaction (cycle 2). More specifically, the product of DNA polymerization reaction in cycle 1 has a specific recognition site and could be cleaved by nicking endonuclease. Then with the help of polymerase in solution, the replication initiated from the cleaving site, which results in strand displacement and is autonomous, forms a large amount of the DNA trigger. The DNA trigger can then hybridize with the remnant MB to show a conformation change, as a “switch” from the hairpin structure to a DNA duplex, resulting in a greater distance between Cy5 and BHQ3 with an increased fluorescence signal. 

### 3.2. The Feasibility of This Strategy

**Figure 1:**
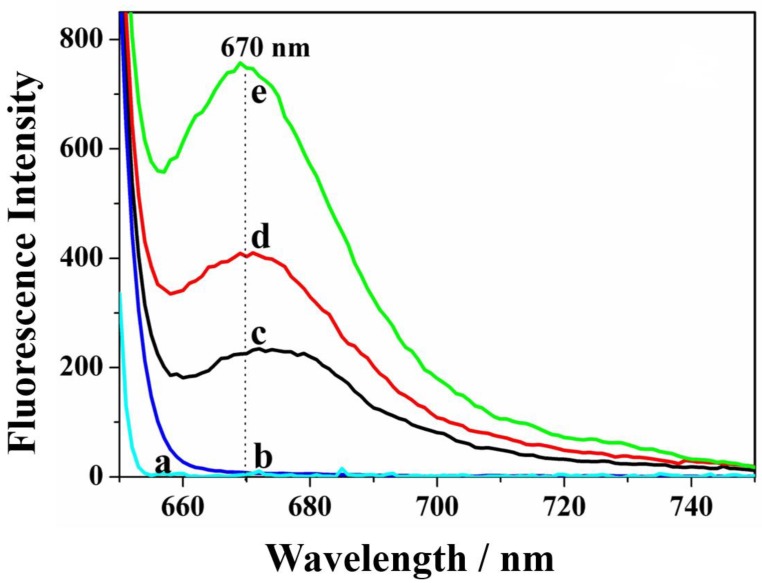
The fluorescence spectrum of the developed sensing system is collected in a blank control sample (curve “a”); in the presence of aptamer DNA, an MB, polymerase, and nicking endonuclease, but without PDGF-BB (curve “b”); in the presence of PDGF-BB, aptamer DNA, and an MB (curve “c”); in the presence of PDGF-BB, aptamer DNA, an MB, and polymerase (curve “d”); and in the presence of PDGF-BB, aptamer DNA, MB, polymerase, and nicking endonuclease Nt.BbvCI (curve “e”). The reaction is in an NEB buffer (pH 7.9) at 37 °C for 2 h containing 1 μM aptamer DNA, 1 μM MB, 2.5 μM primer, 100 ng/mL PDGF-BB, 1 U/μL Klenow Fragment exo-, 2 mM dNTPs, and 1 U/μL Nt.BbvCI.

The primary aspect of our design is whether the two-cycle amplification will work or not, which could be verified by detection of the intensity of fluorescence in different stage directly. As can be seen from curve “a” in Figure 1, the fluorescence signal is hardly observed, indicating that the influence from water is negligible and the background is low in our strategy. In the absence of a target protein, the DNA aptamer exists in their natural secondary structure, which could not hybridize with the MB, and no polymerization reaction is triggered. Therefore, also no obvious fluorescence signal is observed in curve “b”. Curve “c” shows that with the addition of PDGF-BB, the protein induced the conformation switching of the aptamer, which hybridizes with the MB and leads to the open state of MB. Compared with “c”, when the polymerase was spiked in the solution, the enhanced fluorescence signal suggested that the recycling reaction in cycle 1 happened (curve “d”). Subsequently, the introduction of both polymerase and nickase in the solution gives rise to the increment of fluorescence, indicating that the two-stage amplification is successful in the way of our design. At the same concentration of PDGF-BB, the strategy engineered here could produce the highest fluorescence signal for the two-amplification procedure, providing a lower limit of determination and better sensitivity.

### 3.3. Fluorescence Measurement of the Platelet-Derived Growth Factor

**Figure 2:**
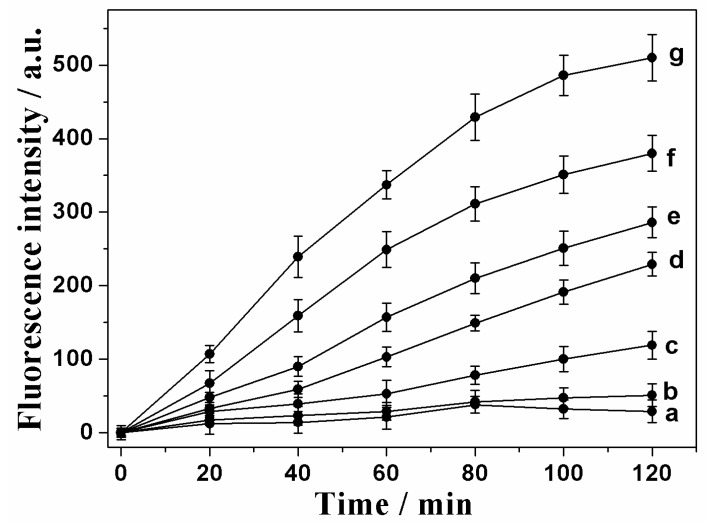
Fluorescence intensity of the proposed aptamer sensing with isothermal circular system containing the DNA aptamer (1 μM), signaling probe (1 μM), primer (2.5 μM), dNTPs (2 mM), Klenow Fragment exo- (1 U/μL), and Nt.BbvCI (1 U/μL), and initiated with a different amount of the PDGF-BB sample (a: 0, b: 0.1 ng/mL, c: 1 ng/mL, d: 10 f ng/mL, e: 20 ng/mL, f: 40 ng/mL, g: 60 ng/mL) at different times.

**Figure 3:**
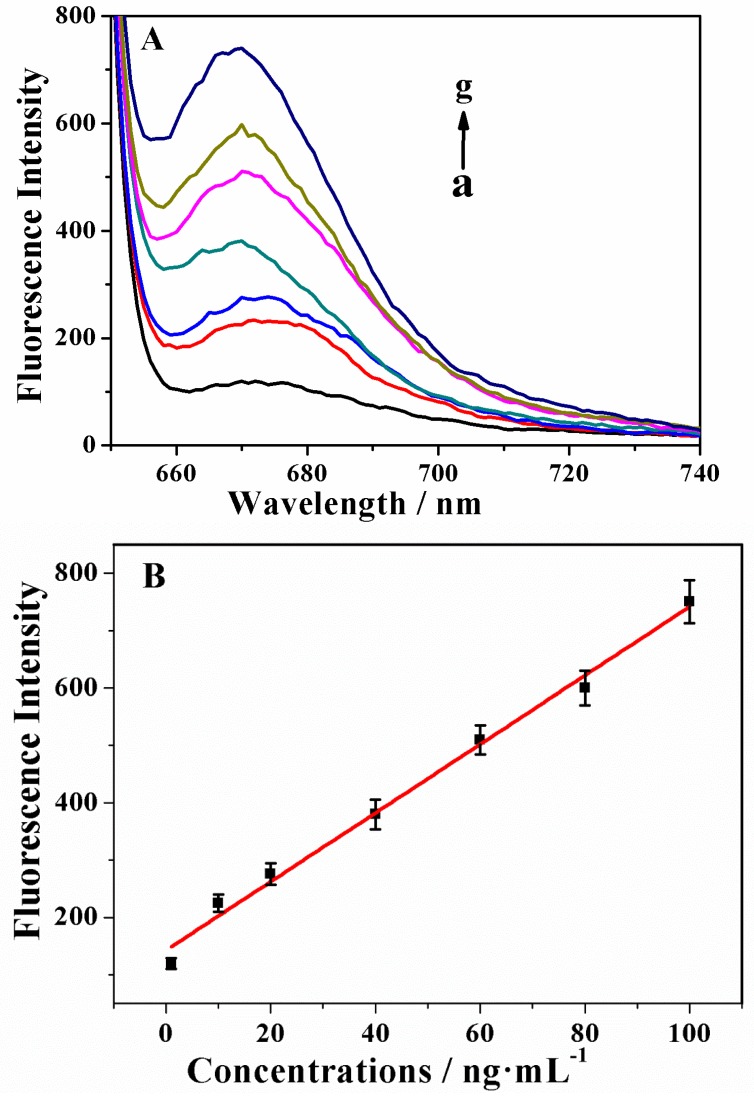
(**A**) Fluorescence spectra of the reaction solution in the presence of different concentrations of PDGF-BB (from “a” to “g”: 1, 10, 20, 40, 60, 80, and 100 ng·mL^−1^) after 2 hours of reaction. (**B**) The linear relationship between the fluorescence peak intensity at 670 nm and the target PDGF-BB concentration. The error bars indicate the standard deviation of repeated three measurements.

The analytical performance of the proposed aptamer sensing could be influenced by the reaction time. From Figure 2, we can see that the fluorescence intensity increased with the increasing reaction time (0–120 min) under different amounts of the PDGF-BB (a: 0, b: 0.1 ng/mL, c: 1 ng/mL, d: 10 ng/mL, e: 20 ng/mL, f: 40 ng/mL, g: 60 ng/mL), using this amplification strategy. At 120 min, the distinction of the fluorescence signal under a different concentration of PDGF-BB was obvious, and the fluorescence intensity was high enough to detect PDGF-BB sensitively. Therefore, 120 min was selected as the reaction time. To evaluate the sensitivity of our new method, different concentrations of PDGF-BB sample were employed, and the blank signal-subtracted fluorescence peak intensity was recorded to find the relationship between the fluorescence results and the concentrations of the target protein. We could find that the fluorescence response increased gradually, with an increase of the target PDGF-BB sample concentration with a linear correlation (Figure 3). The quasi-linear correlation could be depicted by the equation *y* = 5.98*x* + 142.80 (with a correlation coefficient of 0.991, *R^2^* = 0.991), with PDGF-BB sample concentrations ranged from 1.0 to 100 ng·mL^-1^. The limit of detection (LOD), which is defined as three times the standard deviation of blank/slope of calibration curve, is estimated to be 0.74 ng/mL (25 pM, assuming that the molecular weight of PDGF-BB is 30,000 g/mol). The LOD in this signal-on sensing system is superior to that of many other methods, such as the electrochemical measurement [23], colorimetry [24], chemiluminescence assay [25], and electrochemiluminescence assay [26], and is comparable to reported approaches using an DNAzyme [21], hybridization chain reaction [27], or a polymerase-based signal amplification sensor [4]. The obtained ultrahigh sensitivity is mainly because the designed dual signal amplification reactions based on including the aptamer and the oligonucleotide isothermal recycling technique. 

### 3.4. Detection Specificity

**Figure 4:**
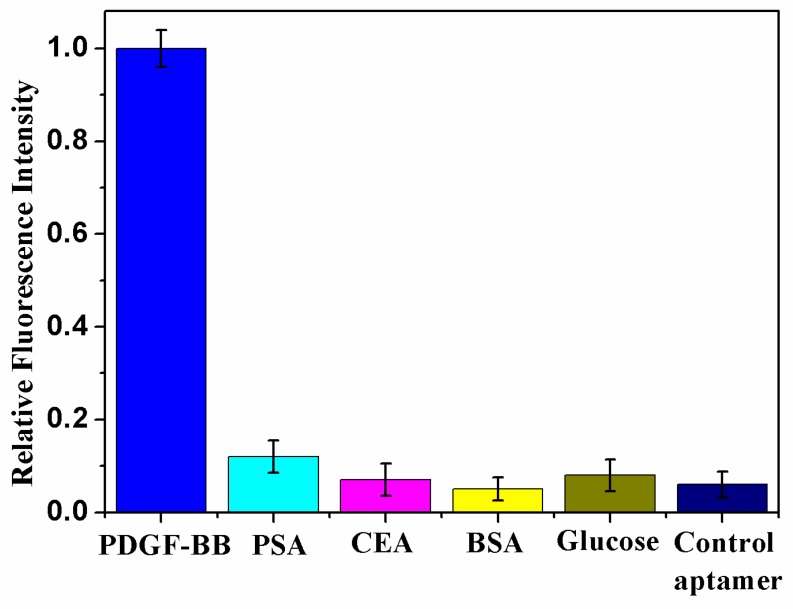
The detection specificity of the proposed aptamer sensor. Prostate-specific antigen (PSA), carcino-embyonic antigen (CEA), albumin from bovine serum (BSA), glucose, and the control aptamer were used to evaluate the detection specificity of the present biosensing system. The concentration of PDGF-BB is 1.0 μg/mL, and the corresponding fluorescence response is defined as 100%. The concentration of the other proteins, such as PSA, CEA, BSA, and glucose is 0.1 mg/mL each. The reaction time is 2 h. The error bars indicate the standard deviation of repeated three measurements.

For an aptamer sensing system, specificity depends on the inherent properties of the selected aptamer matching perfectly with its target original PDGF. Further studies were conducted to find the specificity of this biosensor, using four common interference proteins in human serum (prostate-specific antigen (PSA), carcino-embyonic antigen (CEA), albumin from bovine serum (BSA), and glucose). As Figure 4 showed that the employed four interference proteins displayed no obvious fluorescence response, even in a much higher concentration compared with that of PDGF under the same assay condition. When 1.0 μg/mL of PDGF-BB was added to the reaction system with the same other conditions but using a control aptamer sequence (that does not bind to PDGF-BB), instead of the aptamer sequence of PDGF-BB, it can be seen from Figure 4 that there was also no obvious fluorescence response. The average relative standard deviation (RSD) of the non-specific interaction was 3.28%, suggesting the excellent specificity of this aptamer sensing method for the target PDGF-BB.

### 3.5. Application of the Proposed Biosensor in Human Serum Samples

The proposed aptamer sensing method was evaluated in a complex bio-environment via the recovery test. Fixed concentrations of PDGF-BB protein was spiked in the sample containing 5% BSA and 5% human serum solution, respectively, and then the concentration of the added PDGF-BB protein in the sample was detected, and the recovery of this test was estimated after reaction with the mixture of the PDGF-BB sample at 37 °C for 120 min in the presence of the DNA aptamer (1 μM), signaling probe (1 μM), primer (2.5 μM), dNTPs (2 mM), Klenow Fragment exo- (1 U/μL), NEB buffer, and Nt.BbvCI (1 U/μL). Table 1 showed that the obtained recoveries ranged from 97.0% to 110.4% in 5% BSA or human serum solution, manifesting the excellent performance of this proposed approach in complex media, which is competent in practical clinical application.

## 4. Conclusions

In summary, a smart aptamer sensing method was developed for highly sensitive and selective PDGF-BB assays, through target protein-induced fluorescence changes via DNA molecules conformation changes. Here, combined dual oligonucleotide-based isothermal recycling amplification strategies and highly specific aptamer recognition units, the designed aptamer sensors show a simple but powerful approach without multiple washing steps, as well as precious implementation. This strategy offers a simple, specific and efficient platform to quantify the target protein in low concentrations, and is expected to be applied for other kinds of cancer-related proteins in a convenient way. 

## Figures and Tables

**Table 1:** Results of the recovery test of PDGF-BB in 5% BSA and 5% human serum using the proposed method.

Sample	PDGF-BB Added (ng/mL)	PDGF-BB Found (ng/mL)	Recovery (%)
5% BSA	1	0.97	97
	10	10.06	100.6
	20	20.09	100.5
5% Human Serum	1	1.08	108
	10	11.04	110.4
	20	21.33	106.7
